# Investigation of three alternative histopathological scoring methods at the invasive tumour front in colorectal cancer

**DOI:** 10.1002/2056-4538.70031

**Published:** 2025-05-15

**Authors:** Walaiphorn Woraharn, Ashley McCulloch, Christopher Bigley, Phimmada Hatthakarnkul, Kathryn Pennel, Peter Alexander, Hester van Wyk, Antonia Roseweir, Jennifer Hay, Noori Maka, James Park, Nigel B Jamieson, Joanne Edwards, Campbell SD Roxburgh

**Affiliations:** ^1^ School of Cancer Sciences University of Glasgow Glasgow UK; ^2^ Academic Unit of Surgery University of Glasgow Glasgow UK; ^3^ School of Medicine University of Glasgow Glasgow UK; ^4^ Glasgow Tissue Research Facility Queen Elizabeth University Hospital Glasgow UK; ^5^ Department of Pathology Queen Elizabeth Hospital Glasgow UK; ^6^ Department of Surgery Queen Elizabeth University Hospital Glasgow UK

**Keywords:** histopathological scoring, growth pattern, invasive tumour front, colorectal cancer

## Abstract

Although the characteristics at the invasive tumour front in colorectal cancer (CRC) are simple to assess, they are not included in routine pathology reports because they lack reproducibility and standardisation. In this study, we aimed to validate alternative scoring methods at the invasive tumour front in a large cohort of stage I–III CRC. The retrospective analysis was performed on haematoxylin and eosin–stained sections from 538 patients. At the invasive tumour front, tumour characteristics were scored using three alternative methods: the Karamitopoulou method, which evaluates the percentage of infiltrative tumour; the Taskin method, a five‐point grading scale; and the tumour growth pattern (TGP) method, which classifies patterns as pushing, intermediate, or infiltrative. For interobserver assessment, the Karamitopoulou and TGP methods showed good agreement while the Taskin method presented fair agreement. High scores with the Karamitopoulou and Taskin methods correlated significantly with adverse prognostic factors, particularly advanced T stage (*p* < 0.001), N stage (*p* < 0.001), and the presence of peritoneal involvement (*p* < 0.001). The survival rate of the TGP method demonstrated that patients with an infiltrative growth pattern had significantly worse CRC survival compared to those with pushing and intermediate growth patterns (*p* < 0.001) and the TGP method retained its independence as a prognostic factor in multivariable Cox regression analysis only for colon cancer‐specific survival (*p* < 0.001). The TGP scoring method is an independent prognostic factor only for colon cancer with simple and inexpensive assessment, underlining its practicality in routine reporting. Additionally, this method could be included as an additional histopathological risk indicator with the potential to guide therapeutic decision making.

## Introduction

Colorectal cancer (CRC) is the third most diagnosed cancer and the second leading cause of cancer death in the UK [1]. Up to 44,000 people are diagnosed with the disease annually [2]. Following resection with curative intent for non‐metastatic disease, approximately 40% of patients suffer recurrence and die from their disease [3, 4]. The American Joint Committee on Cancer/the Union for International Cancer Control (AJCC/UICC) established the tumour node metastasis (TNM) classification for prognostication and therapeutic classifications. However, there remains inter‐stage variability of outcomes [5, 6]. Such observations limit the effectiveness of the TNM staging system alone as a prognostic tool. Consequently, refinement of the TNM staging and prediction of outcome after surgical resection is an ongoing priority to ensure patients at higher risk of disease relapse can be considered for additional treatment or enhanced surveillance.

Alternative scoring systems have been proposed since the 1980s when Jass *et al* reported the prognostic value of several additional tumour characteristics. These features include histopathological assessments of invasive tumour front characteristics and lymphocytic infiltrates at the border in rectal cancer [7]. The role of tumour infiltrating lymphocytes has evolved, and the internationally validated Immunoscore has been established as providing additional prognostic information to TNM alone [8, 9, 10]. Various invasive growth pattern assessments have been applied, but the predominant focus has been on tumour budding assessments [11, 12, 13]. This differs from specific assessment of invasive tumour front morphology, which has received less attention in the published literature. Tumour growth pattern (TGP) can be recognised at low magnification and classified as expansile and infiltrative growth patterns in line with Jass' original description in rectal cancer. Several studies demonstrate that the infiltrative growth pattern is associated with poorer cancer outcomes in CRC [14, 15, 16, 17]. Although assessing the invasive front of tumour with H&E staining at low magnification is simple, there is a lack of standardisation and poor reproducibility due to the subjective nature of assessment. These factors limit inclusion in routine pathology reporting. Similar to the original classification by Jass *et al*, Morikawa *et al* utilised a three‐group classification: expansile, intermediate, and infiltrative growth patterns, presenting strong agreement for interobserver assessment (IOA) [18] which has been incorporated into Japanese guidelines for the pathological reporting and classification of CRC [19]. In recent years, two reports from Karamitopoulou and Taskin have proposed novel and structured assessment methods by scaling infiltration at the tumour front in CRC and pancreatic neuroendocrine tumours (PanNETs), respectively. Both studies report improved reproducibility and strong prognostic value from the assessment in the context of routinely reported pathological characteristics. In brief, the Karamitopoulou *et al* method was based on infiltrative tumour border scoring in five percentage increments, whereas the Taskin *et al* method was developed by a 1–5 scaling method depending on characteristics at the border and proportion of clusters [20, 21]. Karamitopoulou observed that the higher infiltrative border score was associated with poorer overall survival (OS), while the Taskin method showed significant prognostic value for PanNETs. Nevertheless, both methods still require validation in external cohorts. Moreover, the Taskin method has not previously been applied in CRC. Thus, we first aimed to externally validate the prognostic value of three infiltrative border scoring methods in a large cohort of primary operable stage I–III CRC patients. Secondly, we aimed to determine which scoring method has the greatest reproducibility between different assessors and the strongest prognostic value. Finally, inter‐relationships between CRC invasive tumour front with established high‐risk pathological characteristics, including those employed in routine reporting, were examined.

## Materials and methods

### Patients and study design

This retrospective study was approved by the West of Scotland Research Ethics Committee (16/WS/0207). All patients provided written informed consent. A total of 787 patients with stage I–III CRC from the Glasgow Royal Infirmary (GRI) between 1997 and 2013 were included. Patients who received neoadjuvant radiotherapy were excluded due to the disruption of tissue architecture that impacts border assessment [22]. The remaining 538 patients were included after removing 30‐day mortality patients. Tumour stage was evaluated by the fifth edition of the AJCC/UICC TNM staging system. To evaluate survival trends over time, 5‐year survival rates were calculated for patients diagnosed during three distinct time periods: 1997–2003, 2004–2008, and 2009–2013.

### Clinicopathological data

Clinical characteristics including age, sex, use of adjuvant therapy, and site of tumour were collected from clinical records. In the UK, the TNM 8 edition has been part of routine pathology reporting (RCPath Colorectal dataset) since 2023. All routinely reported pathological data in this manuscript was in line with RCPath datasets issued at the time of reporting including TNM stage, tumour differentiation, resection margin involvement, venous invasion, perineural invasion, tumour perforation, and peritoneal involvement. Tumour budding was scored previously within this cohort and was defined by International Tumor Budding Consensus Conference 2016 recommendations aligning with the AJCC/UICC 8th edition recommendations for tumour budding evaluation [23, 24]. In brief, tumour budding was evaluated at the invasive tumour front and then classified into two groups: low (0–9 buds) and high (≥10 buds).

### Tumour microenvironment assessments

Klintrup–Mäkinen (KM) grade, tumour stroma percentage (TSP), and the Glasgow Microenvironment Score (GMS) were scored as previously described by Alexander *et al* [25]. In brief, the haematoxylin and eosin (H&E) stained section at the deepest area of invasion at the invasive border was selected for KM scoring, and it was divided into two groups. Low grade indicated absent or weak inflammatory reaction, whereas high grade denoted moderate or prominent inflammation. TSP was determined as low when the stroma percentage was ≤50%, and high if the stroma percentage was >50%. KM and TSP scores were combined to generate a GMS as follows: high KM and any TSP scored GMS 0; low KM and low TSP scored GMS 1; and low KM and high TSP scored GMS 2.

### Assessment of the histopathological growth patterns at the invasive tumour front

#### Karamitopoulou method

**Figure 1:**
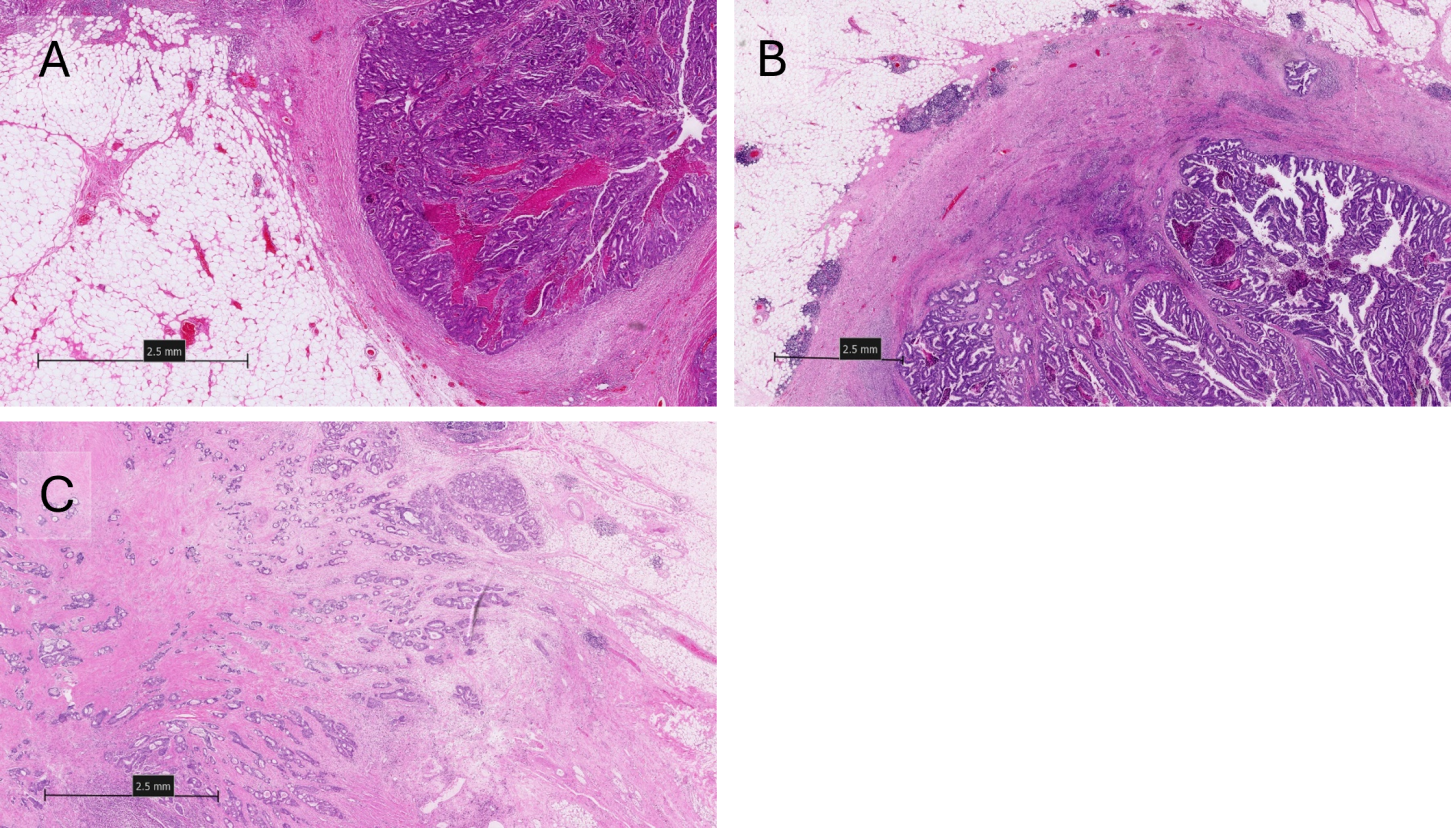
Representative images showing infiltrative components scored by the Karamitopoulou method at different percentages of (A) 0%, (B) 50%, and (C) 100%. Created using NZConnect.

According to the Karamitopoulou method, tumours were scored at the invasive tumour front by infiltrating percentage in 5% increments. In brief, a pushing border was considered 0%, and any areas of infiltration were added to this in 5% increments. The percentage was stratified into two groups: low if the infiltrative border was ≤50%, or high for over 50%. The morphology of the border demonstrating infiltrative growth patterns is represented in Figure 1A–C.

#### Taskin method

**Figure 2:**
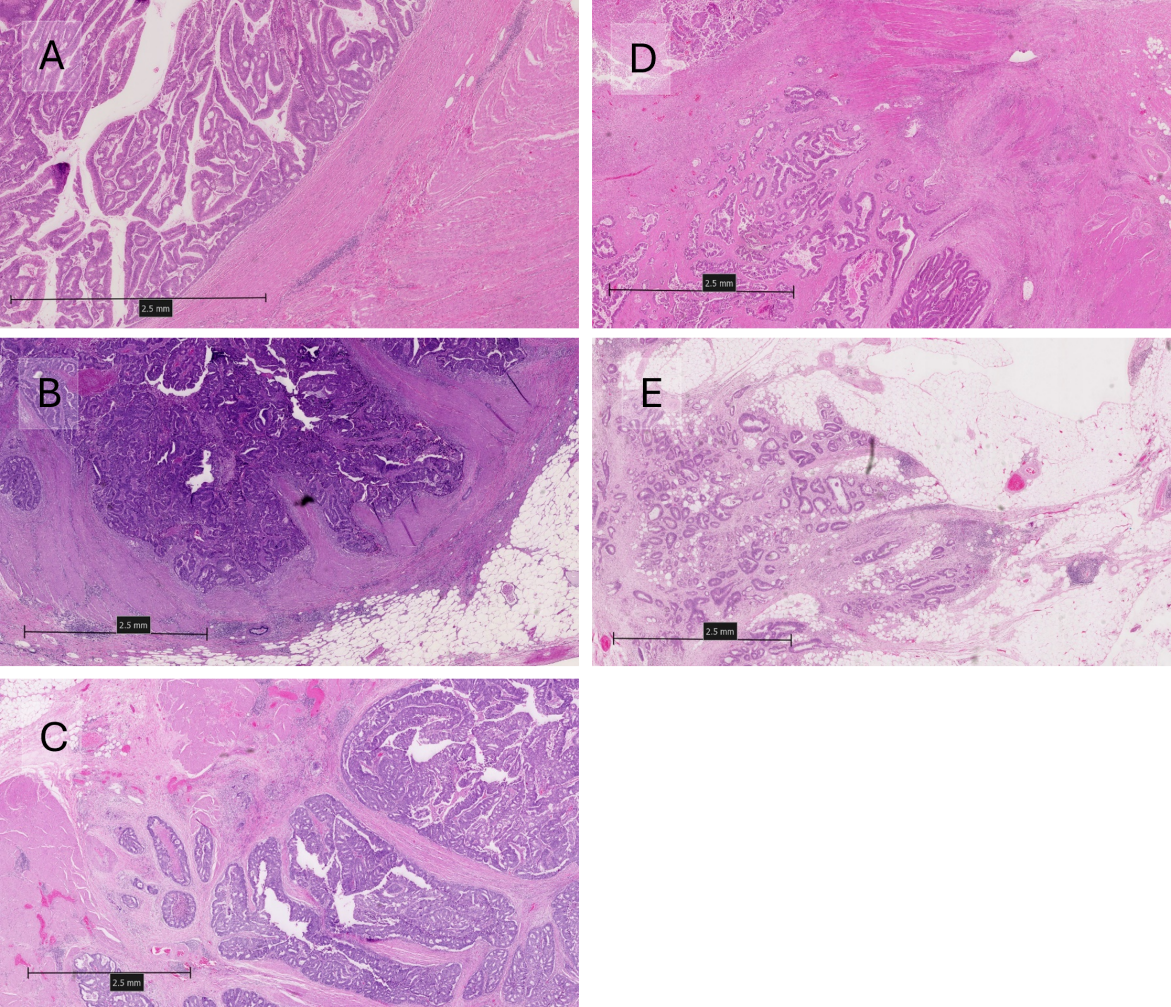
Representative images showing infiltrative components scored by the Taskin method ranging from 1 to 5 points in (A), (B), (C), (D), and (E), respectively. Created using NZConnect.

Tumours were then reviewed using the five‐point scale Taskin method, where score 1 represents well‐circumscribed demarcation; score 2 demonstrates mildly irregular edges with a cluster of cells infiltrating borders near or connected to the parent tumour; score 3 signified large nodules or irregular borders and small clusters spread into peri‐tumoural areas close to the main lesion; score 4 shows small clusters spread more into peri‐tumoural areas but not far from the main tumour; and score 5 denotes lack of tumour border with many clusters located far from the main tumour with expansile infiltration. The tumour infiltrating pattern, according to the five‐point scale, is shown in Figure 2A–E. Scores were then classified into two groups: 1–3 points were defined as low infiltrative, and 4–5 points were deemed as high infiltrative.

#### TGP method

**Figure 3:**
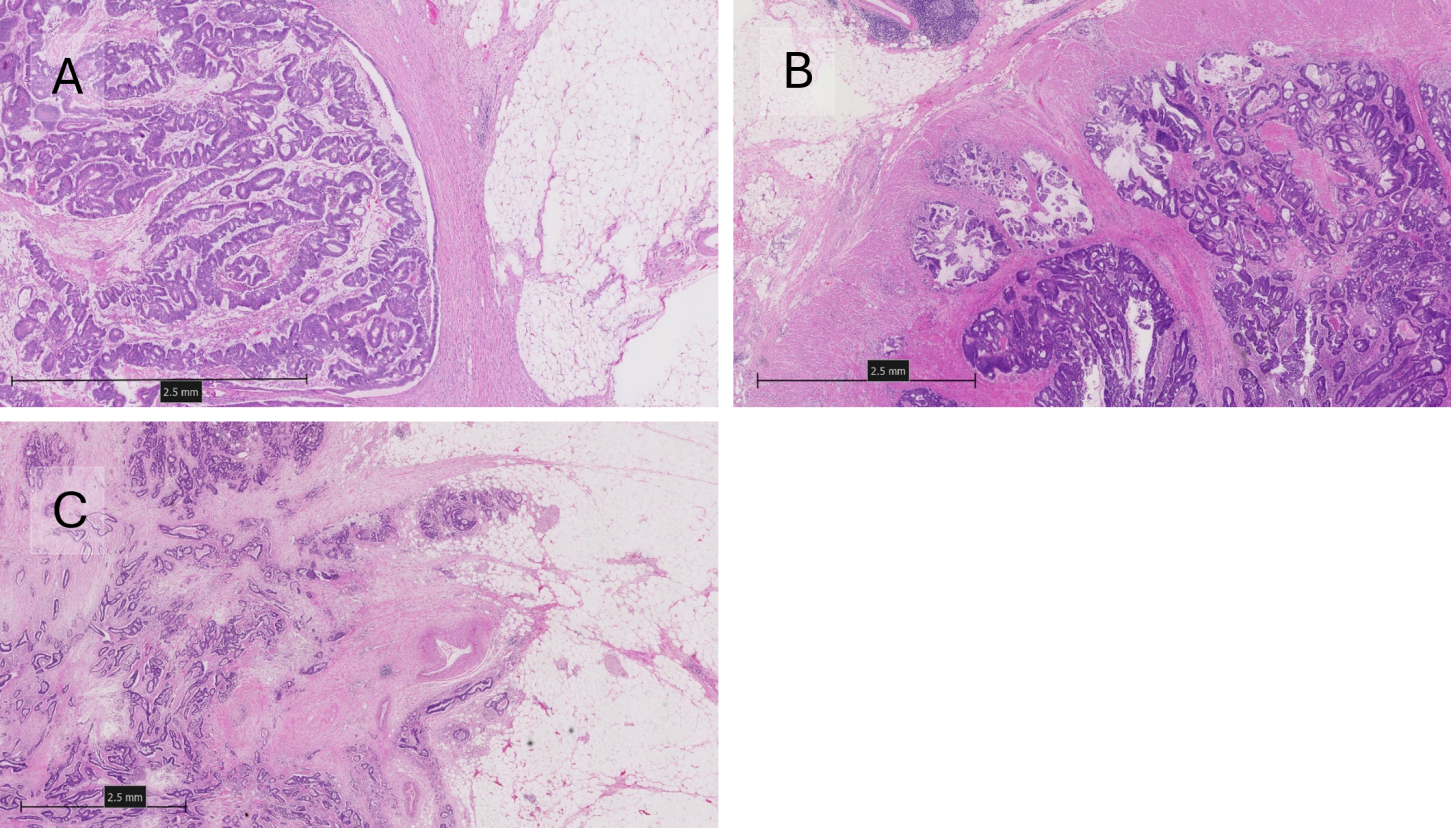
Representative images showing the TGP method, with pushing, intermediate, and infiltrative patterns in (A), (B), and (C), respectively. Created using NZConnect.

TGP was divided into three patterns: pushing, intermediate, and infiltrative growth patterns. Invasive tumour front was evaluated as a ‘pushing’ growth pattern when the border was well circumscribed; ‘intermediate’ growth pattern when the border was not regular, with invasion of medium to large glands; and ‘infiltrative’ growth pattern where small glands invaded diffusely into normal tissues lacking distinct borders [18]. Figure 3A–C shows pushing, intermediate, and infiltrative growth patterns, respectively.

### Interobserver assessment

The H&E‐stained slides from all tumour blocks of 55 patients (10% from total patients) were selected randomly and scored independently by three or two observers (CB, AM, and WW). Each slide represented the highest infiltrative growth pattern at the tumour border of each patient and was scored using the TGP, Karamitopoulou *et al*, and Taskin *et al* scoring methods. The interobserver agreement assessment for the Karamitopoulou method was determined using the intraclass correlation coefficient (ICC). For the Taskin and TGP methods, interobserver agreement was calculated using the weighted kappa.

### Study endpoints

OS was observed from the date of surgery until death or the censoring date. Cancer‐specific survival (CSS) was measured from the surgery date until CRC‐associated death or the censoring date. All survival data were updated to 1 July 2020, which was applied as the censoring date.

### Statistical analysis

Statistical analysis was performed using SPSS version 28.0. The chi‐squared test was performed for categorical data to compare two groups of scores for both scoring methods, and the data shown as frequencies and proportions. OS and CSS curves were computed by the Kaplan–Meier method and compared using the log‐rank test.

Univariate Cox regression analysis was performed to generate hazard ratios (HRs) and 95% confidence intervals (95% CI). Significant variables from the univariate analysis were included in multivariable Cox regression analysis using a backward conditional model to test the statistical independence between infiltrative scoring and clinicopathological variables. The *p* values lower than 0.05 were considered statistically significant.

## Results

### Evaluation of IOA

The ICC value for the Karamitopoulou method reproducibility between observers CB and WW was 0.63, and for AM and WW was 0.79, indicating substantial agreement. The weighted kappa value of the Taskin method for reproducibility between observers CB and WW was 0.31, and for AM and WW was 0.27, indicating fair agreement. The weighted kappa for TGP assessment between CB and WW was 0.78, indicating good agreement. Both Karamitopoulou and TGP presented a similar agreement for interobservers, which was good agreement, and the Taskin method showed fair agreement.

### Survival rates over time period

The 5‐year survival rates were as follows: 65% for 1997–2003, 69% for 2004–2008, and 80% for 2009–2013, indicating an improvement in survival over time.

### Association between clinicopathological characteristics and three alternative histopathological methods

Of the 787 patients, 538 patients, after excluding those who received neoadjuvant therapy or died within 30 days after surgery, were included for three different methods in this study; 56.7% were male and the majority were 50–75 years old (62.3%), had colon cancer (69.5%), and had stage II/III disease (split 52.6% and 47.4%, respectively). The clinicopathological characteristics between groups of the three different methods are reported in Table 1. For the Karamitopoulou method, 190 patients (35.3%) were categorised as low percentage, and 348 (64.7%) were classified as high percentage. For the Taskin method, 176 (32.7%) and 362 (67.3%) were graded as score 1–3 and score 4–5, respectively. Both scoring methods indicate that the higher score groups of infiltrative borders are associated with higher risk clinicopathological characteristics. For the Karamitopoulou method, a higher infiltrative score (51–100%) was associated with increasing T stage (*p* < 0.001) and N stage (*p* < 0.001), stage III CRC (*p* < 0.001), receipt of adjuvant therapy (*p* < 0.001), higher tumour budding (*p* = 0.002), presence of venous invasion (*p* = 0.008), presence of peritoneal involvement (*p* < 0.001), increasing GMS (*p* < 0.001), and higher TSP (*p* < 0.001). For the Taskin method, the higher score group (score 4–5) was associated with increasing T stage (*p* < 0.001) and N stage (*p* < 0.001), stage III CRC (*p* < 0.001), receipt of adjuvant therapy (*p* < 0.001), higher tumour budding (*p* = 0.003), presence of venous invasion (*p* = 0.006), presence of peritoneal involvement (*p* < 0.001), increasing GMS (*p* < 0.001), and higher TSP (*p* < 0.001). There were no statistically significant differences between low and high score groups for both scoring methods when comparing age, sex, differentiation, margin involvement, KM grading, site of tumour, tumour perforation, and period of management.

**Table 1:** Relationship between clinicopathological characteristics and the Karamitopoulou, Taskin, and TGP methods in 538 patients

Features	Total	Karamitopoulou method			Taskin method			TGP method			
		0–50% Infiltrative (*n* = 190)	51–100% Infiltrative (*n* = 348)	*p*	Score 1–3 (*n* = 176)	Score 4–5 (*n* = 362)	*p*	Expansile/pushing (*n* = 168)	Intermediate (*n* = 134)	Infiltrative (*n* = 236)	*p*
Age				0.344			0.516				0.776
<50	37 (6.9)	13 (6.8)	24 (6.9)		11 (6.3)	26 (7.2)		11 (6.5)	11 (8.2)	15 (6.4)	
50–75	335 (62.3)	111 (58.4)	224 (64.4)		105 (59.7)	230 (63.5)		100 (59.5)	82 (61.2)	153 (64.8)	
>75	166 (30.9)	66 (34.7)	100 (28.7)		60 (34.1)	106 (29.3)		57 (33.9)	41 (30.6)	68 (28.8)	
Sex				0.677			0.967				0.853
Female	233 (43.3)	80 (42.1)	153 (44.0)		76 (43.2)	157 (43.4)		70 (41.7)	58 (43.3)	105 (44.5)	
Male	305 (56.7)	110 (57.9)	195 (56.0)		100 (56.8)	205 (56.6)		98 (58.3)	76 (56.7)	131 (55.5)	
T stage				**<0.001**			**<0.001**				**<0.001**
pT1	12 (2.2)	10 (5.3)	2 (0.6)		10 (5.7)	2 (0.6)		10 (6.0)	1 (0.7)	1 (0.4)	
pT2	52 (9.7)	33 (17.4)	19 (5.5)		29 (16.5)	23 (6.4)		28 (16.7)	13 (9.7)	11 (4.7)	
pT3	315 (58.6)	112 (58.9)	203 (58.3)		105 (59.7)	210 (58.0)		99 (58.9)	93 (69.4)	123 (52.1)	
pT4	159 (29.6)	35 (18.4)	124 (35.6)		32 (18.2)	127 (35.1)		31 (18.5)	27 (20.1)	101 (42.8)	
N stage				**<0.001**			**<0.001**				**<0.001**
pN0	308 (57.2)	148 (77.9)	160 (46.0)		137 (77.8)	171 (47.2)		129 (76.8)	82 (61.2)	97 (41.1)	
pN1 and pN2	230 (42.8)	42 (22.1)	188 (54.0)		39 (22.2)	191 (52.8)		39 (23.2)	52 (38.8)	139 (58.9)	
Stage				**<0.001**			**<0.001**				**<0.001**
Stage I	55 (10.2)	37 (19.5)	18 (5.2)		33 (18.8)	22 (6.1)		33 (19.6)	12 (9.0)	10 (4.2)	
Stage II	254 (47.2)	111 (58.4)	143 (41.1)		104 (59.1)	150 (41.4)		96 (57.1)	70 (52.2)	88 (37.3)	
Stage III	229 (42.6)	42 (22.1)	187 (53.7)		39 (22.2)	190 (52.5)		39 (23.2)	52 (38.8)	138 (58.5)	
Site				0.987			0.742				0.420
Colon	374 (69.5)	132 (69.5)	242 (69.5)		124 (70.5)	250 (69.1)		116 (69.0)	99 (73.9)	159 (67.4)	
Rectal	164 (30.5)	58 (30.5)	106 (30.5)		52 (29.5)	112 (30.9)		52 (31.0)	35 (26.1)	77 (32.6)	
Differentiation				0.541			0.948				0.060
Moderate/Well	500 (93.3)	178 (94.2)	322 (92.8)		164 (93.2)	336 (93.3)		157 (94.0)	129 (97.0)	214 (90.7)	
Poor	36 (6.7)	11 (5.8)	25 (7.2)		12 (6.8)	24 (6.7)		10 (6.0)	4 (3.0)	22 (9.3)	
Adjuvant therapy				**<0.001**			**<0.001**				**0.005**
No	331 (70.1)	140 (80.0)	191 (64.3)		128 (80.5)	203 (64.9)		121 (78.6)	83 (71.6)	127 (62.9)	
Received	141 (29.9)	35 (20.0)	106 (35.7)		31 (19.5)	110 (35.1)		33 (21.4)	33 (28.4)	75 (37.1)	
Tumour budding				**0.002**			**0.003**				**<0.001**
Absent	373 (70.8)	146 (79.3)	227 (66.2)		135 (79.4)	238 (66.7)		126 (77.3)	108 (81.8)	139 (59.9)	
Present	154 (29.2)	38 (20.7)	116 (33.8)		35 (20.6)	119 (33.3)		37 (22.7)	24 (18.2)	93 (40.1)	
Margin involvement				0.536			0.513				0.520
Absent	502 (93.3)	179 (94.2)	323 (92.8)		166 (94.3)	336 (92.8)		158 (94.0)	127 (94.8)	217 (91.9)	
Involved	36 (6.7)	11 (5.8)	25 (7.2)		10 (5.7)	26 (7.2)		10 (6.0)	7 (5.2)	19 (8.1)	
Venous invasion				**0.008**			**0.006**				**0.025**
Absent	215 (46.1)	94 (54.0)	121 (41.4)		87 (55.1)	128 (41.6)		80 (52.3)	58 (50.4)	77 (38.9)	
Present	251 (53.9)	80 (46.0)	171 (58.6)		71 (44.9)	180 (58.4)		73 (47.7)	57 (49.6)	121 (61.1)	
Peritoneal involvement				**<0.001**			**<0.001**				**<0.001**
Absent	396 (73.6)	161 (84.7)	235 (67.5)		150 (85.2)	246 (68.0)		143 (85.1)	111 (82.8)	142 (60.2)	
Involved	143 (26.4)	29 (15.3)	113 (32.5)		26 (14.8)	116 (32.0)		25 (14.9)	23 (17.2)	94 (39.8)	
Tumour perforation				0.569			0.176				0.964
0	520 (96.7)	184 (96.8)	336 (96.6)		169 (96.0)	351 (97.0)		163 (97.0)	129 (96.3)	228 (96.6)	
1	12 (2.2)	3 (1.6)	9 (2.6)		3 (1.7)	9 (2.5)		3 (1.8)	3 (2.2)	6 (2.5)	
2	6 (1.1)	3 (1.6)	3 (0.9)		4 (2.3)	2 (0.6)		2 (1.2)	2 (1.5)	2 (0.8)	
GMS				**<0.001**			**<0.001**				**<0.001**
0	94 (17.9)	39 (21.2)	55 (16.1)		34 (20.0)	60 (16.9)		33 (20.4)	23 (17.3)	38 (16.5)	
1	301 (57.2)	129 (70.1)	172 (50.3)		120 (70.6)	181 (50.8)		117 (72.2)	80 (60.2)	104 (45.0)	
2	131 (24.9)	16 (8.7)	115 (33.6)		16 (9.4)	115 (32.3)		12 (7.4)	30 (22.6)	89 (38.5)	
KM				0.144			0.378				0.595
Low grade	432 (82.1)	145 (78.8)	287 (83.9)		136 (80.0)	296 (83.1)		129 (79.6)	110 (82.7)	193 (83.5)	
High grade	94 (17.9)	39 (21.2)	55 (16.1)		34 (20.0)	60 (16.9)		33 (20.4)	23 (17.3)	38 (16.5)	
TSP				**<0.001**			**<0.001**				**<0.001**
Low	380 (72.2)	165 (89.7)	215 (62.9)		153 (90.0)	227 (63.8)		148 (91.4)	99 (74.4)	133 (57.6)	
High	146 (27.8)	19 (10.3)	127 (37.1)		17 (10.0)	129 (36.2)		14 (8.6)	34 (25.6)	98 (42.4)	
Time period				0.169			0.225				0.219
1997–2003	138 (29.6)	45 (25.9)	93 (31.8)		43 (27.2)	95 (30.8)		38 (24.8)	35 (30.4)	65 (32.8)	
2004–2008	144 (30.9)	51 (29.3)	93 (31.8)		44 (27.8)	100 (32.5)		45 (29.4)	33 (28.7)	66 (33.3)	
2009–2013	184 (39.5)	78 (44.8)	106 (36.3)		71 (44.9)	113 (36.7)		70 (45.8)	47 (40.9)	67 (33.8)	
MMR status				0.122			**0.048**				0.459
dMMR	149 (32.5)	62 (36.9)	87 (29.9)		59 (38.6)	90 (29.4)		51 (34.7)	32 (27.8)	66 (33.5)	
pMMR	310 (67.5)	106 (63.1)	204 (70.1)		94 (61.4)	216 (70.6)		96 (65.3)	83 (72.2)	131 (66.5)	

Bold *p* values are statistically significant.

For the TGP method, among 538 patients with CRC, 168 patients (31.2%) had tumours with a pushing pattern, 134 (24.9%) exhibited the intermediate pattern, and 236 (43.9%) showed the infiltrative pattern. Table 1 demonstrates the relationships between clinicopathological features and TGP. Infiltrative growth pattern was associated with histopathological features of an adverse prognosis: higher T stage (*p* < 0.001), higher N stage (*p* < 0.001), receipt of adjuvant therapy (*p* = 0.005), presence of tumour budding (*p* < 0.001), presence of venous invasion (*p* = 0.025), presence of peritoneal involvement (*p* < 0.001), higher GMS (*p* < 0.001), and higher TSP (*p* < 0.001). On the other hand, age, sex, site of tumour, margin involvement, tumour perforation, and KM showed no association with TGP (*p* > 0.05).

### Survival and Cox regression analysis

**Figure 4:**
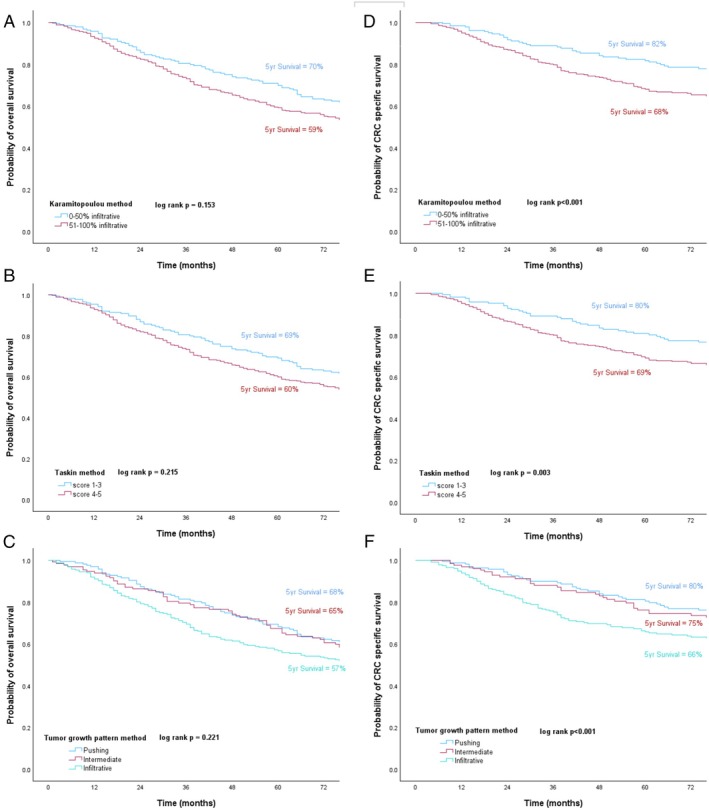
Kaplan–Meier curves for CRC OS for (A) Karamitopoulou, (B) Taskin, and (C) TGP methods, and CRC‐specific survival for (D) Karamitopoulou, (E) Taskin, and (F) TGP methods.

Table 2 shows the univariate and multivariable Cox regression survival analysis for OS in all patients with CRC included in this study. The median follow‐up for survivors was 91 months. Univariate analysis showed that parameters significantly related to OS included age, T stage, N stage, margin involvement, venous invasion, peritoneal involvement, tumour budding, GMS, KM, TSP, and time period. The Karamitopoulou method, the Taskin method, and TGP assessment were not found to be related to OS. On multivariable analysis for OS, age [HR = 2.76 (2.19–3.49), *p* < 0.001], T stage [HR = 1.41 (1.16–1.72), *p* = 0.001], margin involvement [HR = 2.29 (1.44–3.64), *p* < 0.001], and tumour budding [HR = 1.35 (1.05–1.72), *p* = 0.019] retained independent association with OS. Figure 4A–C showed the Kaplan–Meier survival curves for the Karamitopoulou method (*p* = 0.153), the Taskin method (*p* = 0.215), and the TGP (*p* = 0.221) method, respectively.

**Table 2:** Cox regression analysis for colorectal cancer overall survival in 538 patients

Features	Univariate HR (95% CI)	*p*	Multivariable HR (95% CI)	*p*
Age (<50/50–75/>75)	2.40 (1.96–2.93)	**<0.001**	2.76 (2.19–3.49)	**<0.001**
Sex (Female/Male)	1.02 (0.82–1.27)	0.857		
T stage (pT1/pT2/pT3/pT4)	1.43 (1.21–1.69)	**<0.001**	1.41 (1.16–1.72)	**<0.001**
N stage (pN0/pN1 and pN2)	1.30 (1.05–1.62)	**0.015**	1.07 (0.83–1.38)	0.593
Site (Colon/Rectum)	0.96 (0.76–1.21)	0.711		
Differentiation (Moderate and Well/Poor)	1.04 (0.67–1.60)	0.873		
Margin involvement (Absent/Involved)	2.21 (1.52–3.22)	**<0.001**	2.29 (1.44–3.64)	**<0.001**
Venous invasion (Absent/Present)	1.41 (1.11–1.78)	**0.004**	1.21 (0.95–1.56)	0.128
Peritoneal involvement (Absent/Involved)	1.49 (1.18–1.88)	**<0.001**	1.04 (0.68–1.59)	0.861
Tumour perforation (0/1/2)	1.32 (0.90–1.94)	0.162		
Tumour budding (Absent/Present)	1.33 (1.06–1.67)	**0.015**	1.35 (1.05–1.72)	**0.019**
GMS (0/1/2)*	1.45 (1.22–1.72)	**<0.001**		
KM (Low/High)	0.64 (0.48–0.87)	**0.005**	0.79 (0.55–1.14)	0.214
TSP (Low/High)	1.44 (1.14–1.82)	**0.003**	1.25 (0.94–1.66)	0.121
Karamitopoulou method (0–50%/51–100%)	1.18 (0.94–1.48)	0.155		
Taskin method (0–3/4–5)	1.16 (0.92–1.45)	0.217		
TGP method (Pushing/Intermediate/Infiltrative)	1.12 (0.98–1.26)	0.088		
MMR status (dMMR/pMMR)	0.89 (0.70–1.14)	0.357		
Time period (1997–2003/2004–2008/2009–2013)	0.84 (0.72–0.97)	**0.016**	0.88 (0.75–1.03)	0.103

Bold *p* values are statistically significant.

* Not included in the multivariable analysis due to multicollinearity.

Table 3 shows the univariate and multivariable Cox regression survival analysis for CSS for all CRC patients. On univariate analysis, age, T stage, N stage, margin involvement, venous invasion, peritoneal involvement, tumour perforation, tumour budding, GMS, KM, TSP, time period, the Karamitopoulou method, the Taskin method, and TGP method were significantly related to colorectal CSS. On multivariable analysis, age [HR = 1.86 (1.34–2.58), *p* < 0.001], T stage [HR = 1.48 (1.09–2.01), *p* = 0.011], N stage [HR = 1.54 (1.07–2.21), *p* = 0.020], margin involvement [HR = 4.20 (2.50–7.04), *p* < 0.001], and tumour budding [HR = 1.82 (1.28–2.58), *p* < 0.001] were independently associated with colorectal CSS. Figure 4D,E show the Kaplan–Meier survival curves for colorectal CSS using the Karamitopoulou method (*p* < 0.001) and the Taskin method (*p* = 0.003); higher scores from both methods were significantly associated with shorter CRC survival rates. Figure 4F shows Kaplan–Meier curves of the TGP method indicating that the infiltrative growth pattern had the worst CRC survival rate compared with pushing and intermediate growth patterns (*p* < 0.001).

**Table 3:** Cox regression analysis for colorectal cancer‐specific survival in 538 patients

Features	Univariate HR (95% CI)	*p*	Multivariable HR (95% CI)	*p*
Age (<50/50–75/>75)	1.47 (1.12–1.94)	**0.006**	1.86 (1.34–2.58)	**<0.001**
Sex (Female/Male)	1.19 (0.87–1.62)	0.277		
T stage (pT1/pT2/pT3/pT4)	2.17 (1.68–2.80)	**<0.001**	1.48 (1.09–2.01)	**0.011**
N stage (pN0/pN1 and pN2)	1.92 (1.42–2.60)	**<0.001**	1.54 (1.07–2.21)	**0.020**
Site (Colon/Rectum)	0.79 (0.57–1.11)	0.181		
Differentiation (Moderate and Well/Poor)	1.20 (0.67–2.16)	0.545		
Margin involvement (Absent/Involved)	4.32 (2.86–6.54)	**<0.001**	4.20 (2.50–7.04)	**<0.001**
Venous invasion (Absent/Present)	1.60 (1.14–2.24)	**0.007**	1.23 (0.84–1.82)	0.285
Peritoneal involvement (Absent/Involved)	2.20 (1.61–2.99)	**<0.001**	1.09 (0.59–2.02)	0.788
Tumour perforation (0/1/2)	1.68 (1.06–2.67)	**0.028**	0.90 (0.31–2.60)	0.850
Tumour budding (Absent/Present)	1.87 (1.37–2.55)	**<0.001**	1.82 (1.28–2.58)	**<0.001**
GMS (0/1/2)*	2.09 (1.63–2.67)	**<0.001**		
KM (Low/High)	0.31 (0.17–0.55)	**<0.001**	0.57 (0.29–1.09)	0.090
TSP (Low/High)	1.89 (1.38–2.60)	**<0.001**	1.15 (0.77–1.70)	0.495
Karamitopoulou method (0–50%/51–100%)	1.85 (1.30–2.62)	**<0.001**	0.94 (0.30–2.96)	0.913
Taskin method (0–3/4–5)	1.69 (1.19–2.41)	**0.004**	0.85 (0.41–1.76)	0.667
TGP method (Pushing/Intermediate/Infiltrative)	1.42 (1.18–1.72)	**<0.001**	1.23 (0.98–1.54)	0.079
MMR status (dMMR/pMMR)	0.93 (0.65–1.33)	0.692		
Time period (1997–2003/2004–2008/2009–2013)	0.76 (0.62–0.93)	**0.008**	0.89 (0.72–1.10)	0.266

Bold *p* values are statistically significant.

* Not included in the multivariable analysis due to multicollinearity.

**Figure 5:**
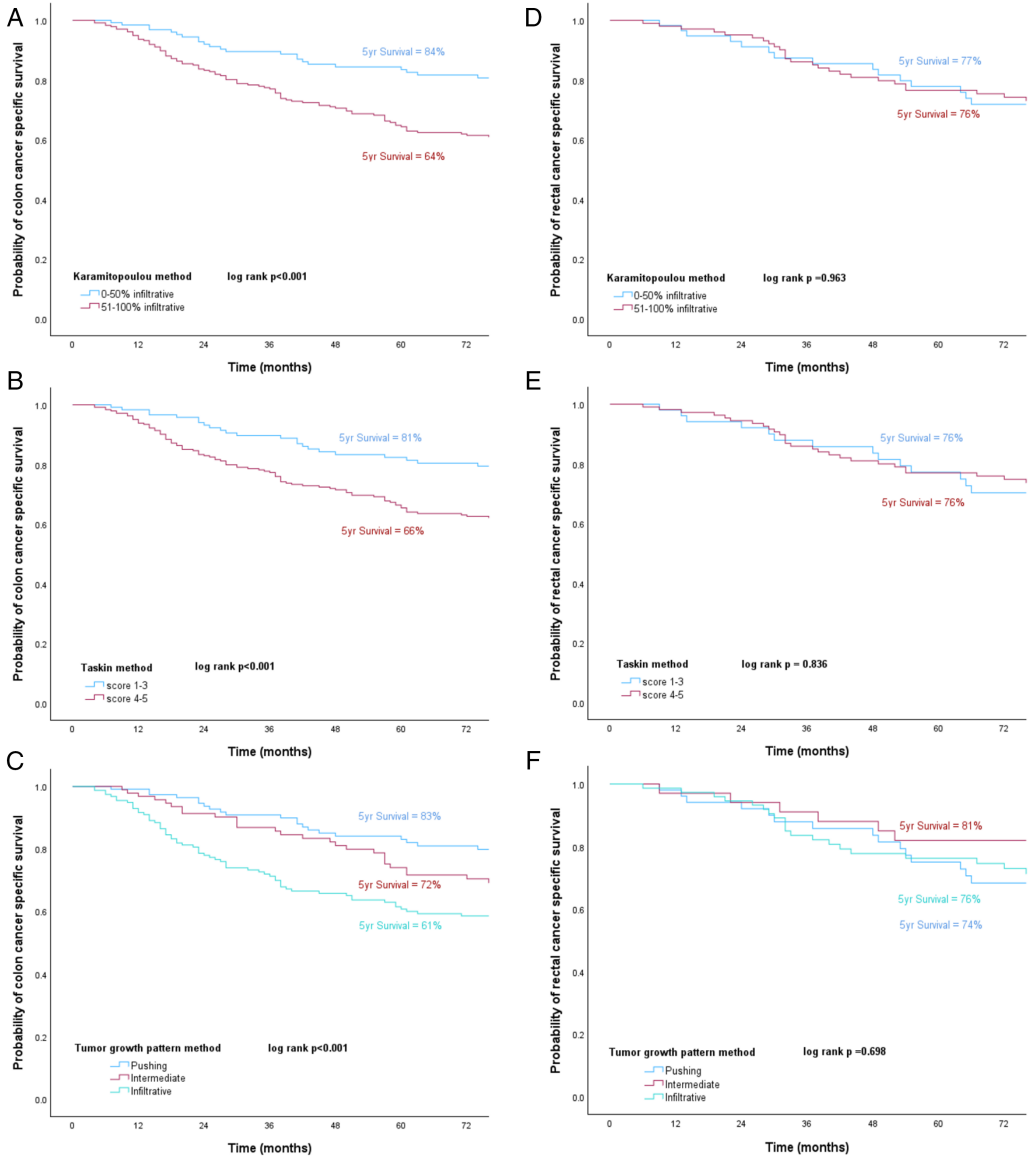
Kaplan–Meier curves for colon cancer CSS for (A) Karamitopoulou, (B) Taskin, and (C) TGP methods, and rectal cancer CSS for (D) Karamitopoulou, (E) Taskin, and (F) TGP methods.

Table 4 shows the univariate and multivariable Cox regression survival analysis for CSS of colon cancer patients. On univariate analysis, age, T stage, N stage, margin involvement, venous invasion, peritoneal involvement, tumour perforation, tumour budding, GMS, KM, TSP, time period, the Karamitopoulou method, the Taskin method, and the TGP method were significantly related to colon CSS. On multivariable analysis, age [HR = 2.80 (1.89–4.14), *p* < 0.001], T stage [HR = 2.11 (1.40–3.17), *p* < 0.001], margin involvement [HR = 4.99 (2.46–10.13), *p* < 0.001], and TGP method [HR = 1.69 (1.27–2.24), *p* < 0.001] retained independence. Figure 5A–C show the Kaplan–Meier curves for colon CSS using the Karamitopoulou method (*p* < 0.001), the Taskin method (*p* < 0.001), and the TGP categories (*p* < 0.001).

**Table 4:** Cox regression analysis for colon cancer‐specific survival in 374 patients

Features	Univariate HR (95% CI)	*p*	Multivariable HR (95% CI)	*p*
Age (<50/50–75/>75)	1.83 (1.32–2.53)	**<0.001**	2.83 (1.91–4.20)	**<0.001**
Sex (Female/Male)	1.06 (0.74–1.52)	0.751		
T stage (pT1/pT2/pT3/pT4)	2.64 (1.92–3.63)	**<0.001**	2.11 (1.40–3.19)	**<0.001**
N stage (pN0/pN1 and pN2)	2.03 (1.42–2.90)	**<0.001**	1.32 (0.86–2.04)	0.201
Differentiation (Moderate and Well/Poor)	1.35 (0.73–2.52)	0.339		
Margin involvement (Absent/Involved)	4.99 (2.94–8.47)	**<0.001**	4.60 (2.27–9.32)	**<0.001**
Venous invasion (Absent/Present)	1.60 (1.06–2.40)	**0.024**	1.27 (0.79–2.03)	0.327
Peritoneal involvement (Absent/Involved)	2.63 (1.85–3.75)	**<0.001**	1.23 (0.56–2.71)	0.610
Tumour perforation (0/1/2)	1.66 (1.05–2.63)	**0.031**	0.82 (0.25–2.66)	0.740
Tumour budding (Absent/Present)	1.59 (1.10–2.30)	**0.013**	1.32 (0.86–2.04)	0.204
GMS (0/1/2)*	2.15 (1.60–2.87)	**<0.001**		
KM (Low/High)	0.32 (0.16–0.64)	**0.001**	0.52 (0.24–1.13)	0.099
TSP (Low/High)	2.11 (1.46–3.04)	**<0.001**	0.93 (0.58–1.50)	0.772
Karamitopoulou method (0–50%/51–100%)	2.41 (1.55–3.73)	**<0.001**	1.18 (0.32–4.42)	0.801
Taskin method (0–3/4–5)	2.19 (1.41–3.39)	**<0.001**	0.72 (0.30–1.72)	0.456
TGP method (Pushing/Intermediate/Infiltrative)	1.68 (1.34–2.12)	**<0.001**	1.65 (1.24–2.18)	**<0.001**
MMR status (dMMR/pMMR)	1.00 (0.67–1.51)	0.985		
Time period (1997–2003/2004–2008/2009–2013)	0.68 (0.53–0.86)	**0.002**	0.78 (0.61–1.01)	0.056

Bold *p* values are statistically significant.

* Not included in the multivariable analysis due to multicollinearity.

Table 5 shows the univariate and multivariable Cox regression survival analysis for CSS of rectal cancer patients. On univariate analysis, margin involvement (HR = 4.34 [2.20–8.57], *p* < 0.001), tumour budding (HR = 2.86 [1.58–5.18], *p* < 0.001), GMS (HR = 1.94 [1.22–3.09], *p* = 0.005), and KM (HR = 0.27 [0.08–0.88], *p* = 0.029) increased the risk of rectal cancer‐caused mortality, while the multivariable analysis demonstrated that margin involvement (HR = 2.80 [1.29–6.07], *p* = 0.009), tumour budding (HR = 2.22 [1.19–4.15], *p* = 0.012), and GMS (HR = 1.69 [1.03–2.77], *p* = 0.038) retained independence. Figure 5D–F show the Kaplan–Meier survival curves indicating rectal CSS for the Karamitopoulou method (*p* = 0.963), the Taskin method (*p* = 0.836), and TGP method (*p* = 0.698).

**Table 5:** Cox regression analysis for rectal cancer‐specific survival in 164 patients

Features	Univariate HR (95% CI)	*p*	Multivariable HR (95% CI)	*p*
Age (<50/50–75/>75)	0.78 (0.45–1.34)	0.368		
Sex (Female/Male)	1.69 (0.90–3.16)	0.103		
T stage (pT1/pT2/pT3/pT4)	1.34 (0.85–2.12)	0.214		
N stage (pN0/pN1 and pN2)	1.67 (0.94–2.99)	0.082		
Differentiation (Moderate and Well/Poor)	0.46 (0.06–3.37)	0.448		
Margin involvement (Absent/Involved)	4.34 (2.20–8.57)	**<0.001**	2.80 (1.29–6.07)	**0.009**
Venous invasion (Absent/Present)	1.63 (0.87–3.05)	0.129		
Peritoneal involvement (Absent/Involved)	0.82 (0.32–2.07)	0.670		
Tumour budding (Absent/Present)	2.86 (1.58–5.18)	**<0.001**	2.22 (1.19–4.15)	**0.012**
GMS (0/1/2)	1.94 (1.22–3.09)	**0.005**	1.69 (1.03–2.77)	**0.038**
KM (Low/High)	0.27 (0.08–0.88)	**0.029**	0.64 (0.15–2.79)	0.549
TSP (Low/High)	1.47 (0.79–2.72)	0.223		
Karamitopoulou method (0–50%/51–100%)	1.01 (0.55–1.86)	0.963		
Taskin method (0–3/4–5)	0.94 (0.51–1.74)	0.837		
TGP method (Pushing/Intermediate/Infiltrative)	0.98 (0.70–1.36)	0.896		
MMR status (dMMR/pMMR)	0.81 (0.39–1.65)	0.555		
Time period (1997–2003/2004–2008/2009–2013)	0.98 (0.68–1.40)	0.906		

Bold *p* values are statistically significant.

**Figure 6:**
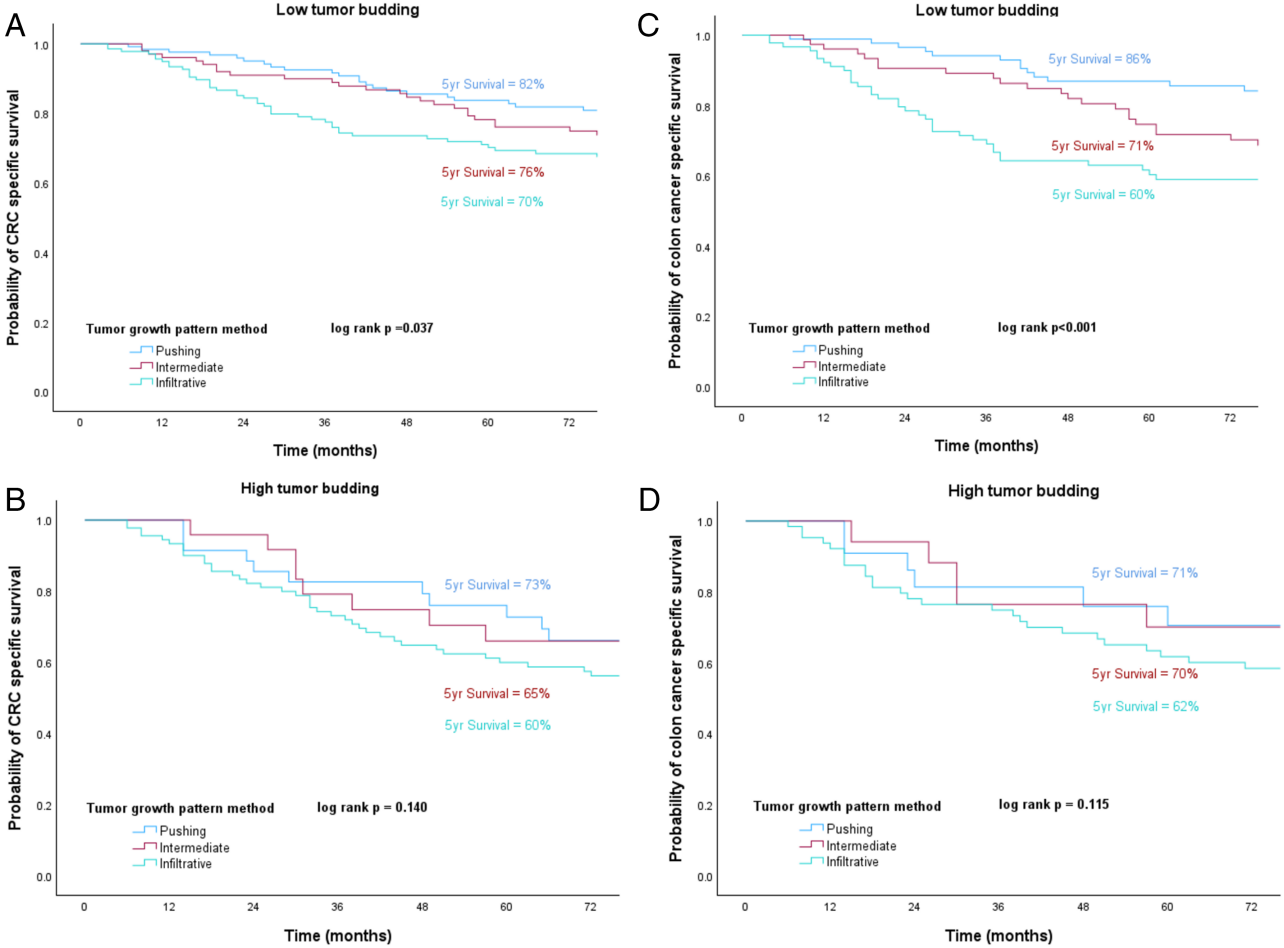
Kaplan–Meier curves for CRC CSS for the TGP method stratified by (A) low and (B) high tumour budding. Kaplan–Meier curves for colon cancer CSS for the TGP method stratified by (C) low and (D) high tumour budding.

When stratified by tumour budding, the TGP method demonstrated significant survival differences among growth patterns in low tumour budding patients, with the infiltrative growth pattern associated with a worse prognosis and the pushing pattern with a better prognosis. However, in high tumour budding patients, they did not show significant survival differences. These findings were consistent for all CRC and for colon cancer specifically (Figure 6).

## Discussion

Given the variation in outcomes for patients with stage II and stage III CRC, there remains a need for refinement of outcome prediction to ensure adjuvant therapy and surveillance can be optimally planned. We report a strong association between three tumour border assessment tools and CSS in a large CRC cohort. Importantly, we have validated the Taskin and Karamitopoulou assessments as conferring prognostic value. However, in our full cohort analysis, these features were not independent of tumour budding in their association with CSS. While time period and MMR status affect the treatment, multivariable Cox regression analysis did not show that they had independent significance. This suggests that survival outcomes of primary CRC are driven by tumour stage, age, margin involvement, and TGP rather than time period or MMR status. When colon cancer was considered alone, tumour border characteristics did retain stage and tumour budding‐independent association with CSS. The results highlight that the simplicity of the traditional scoring method can be utilised as a prognostic factor for colon cancer alone. Previous studies have demonstrated that patients whose tumours had an infiltrative growth pattern had shorter survival when compared to those with a pushing growth pattern [14, 17, 18]. In this study, we demonstrated the prognostic value of the Taskin method in CRC for the first time, having previously been shown to have utility in PanNETs. We adapted the Taskin method by utilising only the microscopic infiltrative pattern criteria from one slide with the highest score. We also found that a higher score with the Taskin method was associated with adverse outcomes, including higher T and N stage, in common with other border assessment scoring methods. In addition, Karamitopoulou demonstrated that a high infiltrative pattern was associated with adverse clinical outcomes which correlate with our results [20]. Several studies have previously confirmed that the infiltrative growth pattern is associated with worse clinical outcomes [14, 17, 18]. Our study is the first to report all three border assessments in the same cohort. Compared to the Taskin and TGP methods, Karamitopoulou assessment is the most complex and time‐consuming assessment as it is defined by 5% increments. Regarding reproducibility, the Karamitopoulou and TGP methods showed strong agreement; however, the TGP method is more easily and quickly assessed because it uses categorical scoring.

Each of these border assessments, including tumour budding, represents a continuum of the same biological phenomenon indicating morphological aggressiveness. Biologically, the invasive edge represents the interaction between tumour‐related factors and the host. Koelzer and Lugli suggest that the tumour border is an area of tumour cells that has directly migrated into host tissue after division; the infiltrative growth pattern represents directed tumour growth, whereas the pushing growth pattern embodies non‐directed tumour growth or random arrangement of tumour cells [26]. For the molecular alteration aspect, the infiltrative growth pattern was found to be related to *BRAF* mutation, whereas the pushing pattern was associated with microsatellite instability (MSI) status (high MSI) [18, 26]. In addition, E‐cadherin (CDH1) is a protein that is associated with tumour invasiveness and tumour progression [27, 28]. Loss of CDH1 expression has been associated with poor prognosis in CRC in other studies [29]. Kim *et al* have shown that the loss of CDH1 expression is associated with the infiltrative growth pattern [30]. A recent study has revealed that CRC migration occurs along enteric neurons partly via L1CAM and N‐cadherin supporting long and fast invasion of tumour cells [31]. Moreover, Rajaganeshan *et al* described how the infiltrative growth pattern of primary CRC might express high hypoxia inducible factor (HIF‐1α) leading to tumour invasion via MMP protease upregulation [32]. In oesophageal cancer, epithelial to mesenchymal transition (EMT), angiogenesis, and inflammation pathways were enriched in the infiltrative growth pattern, concordant with cancer‐associated fibroblast upregulation compared with the expansile growth pattern [33].

One of the potential mechanisms underlying the infiltrative growth pattern involves tumour cell invasion into surrounding tissue via dysregulation of the Wnt signalling pathway, an important protein interaction network. Wnt signalling contributes to cytoplasmic β‐catenin stabilisation. Around 80% of CRC cases have adenomatous polyposis coli (*APC*) mutation, a tumour suppressor gene, leading to the accumulation of nuclear β‐catenin and increased expression of target genes associated with proliferation and invasion [34]. Studies have revealed that increased expression of nuclear β‐catenin was associated with tumour budding at the invasive edge in CRC [35, 36]. Some studies showed that Wnt/β‐catenin activation can induce EMT in CRC [37, 38]. EMT is a biological process that increases cell mobility. It alters between epithelial and mesenchymal phases, causing cell migration and invasion. The processes of EMT activation include cell–cell interaction, cytoskeleton remodelling, and alteration of EMT markers such as decreased expression of CDH1, increased expression of N‐cadherin and vimentin, including EMT‐inducible transcription factors such as SNAIL, SLUG, and TWIST. For instance, an increase in Wnt signalling results in the upregulation of SNAIL that plays a role in CDH1 suppression, leading to migration, local invasion, and EMT regulation [39]. Interestingly, ZEB2 is found at the invasive tumour front of CRC and induces EMT by matrix metalloproteinase (MMP) upregulation that can promote basement membrane breakdown, supporting cancer cell invasion [40, 41].

Tumour budding is a feature observed in a subgroup of CRC patients at the tumour border defined as clusters of undifferentiated tumour cells. It is also different from tumour border growth pattern assessment as it is recognised under high magnification whereas TGP evaluation can be made at low magnification. Although high tumour budding can be found in infiltrative growth patterns, the Karamitopoulou data show that tumour budding does not need to be applied to differentiate between pushing and infiltrative growth patterns [20]. High tumour budding is an established and validated negative prognostic feature, associated with poorer oncological outcomes in CRC [42, 43, 44, 45]. In this study, high tumour budding was a prognostic factor when compared to other border morphological assessments in all CRC and rectal cancer in multivariable analysis. Furthermore, TGP seemed to offer additional prognostic value in patients with low budding tumours since this method showed significantly different survival outcomes between TGP categories in patients with low budding in both CRC and colon cancer. Nevertheless, when multivariable Cox regression was applied, TGP was an independent prognostic factor only in colon cancer and not in CRC, reflecting biological differences underlying colon and rectal cancers. Future studies need to further define why the prognostic independence of TGP is prominent exclusively in colon cancer, potentially by investigating distinct molecular mechanisms.

The three different assessments at the invasive tumour front were established using approaches that differed in their evaluation scales: the Karamitopoulou method is scored from 0% to 100% infiltrative growth, the Taskin classification employs a score from 1 to 5 based on infiltrative pattern, and the TGP method uses trichotomous (providing more detail than dichotomous) classification. These approaches have reduced the classification uncertainty, such as tumours exhibiting an intermediate growth pattern, when compared to the traditional dichotomous scoring method created by Jass *et al* [7]. Additionally, the interobserver agreement was good for both the Karamitopoulou and TGP methods, indicating high reproducibility. The robustness of this study lies in analysing a large‐scale CRC cohort, which includes the complete clinical data set for 538 patients and adapting the Taskin scoring method for CRC. Moreover, we evaluated the three alternative scoring systems in colon, rectal, and all CRC separately. We found that, in rectal cancer patients, there were no differences in HR for the three different methods between score groups. However, with only 45 rectal cancer deaths included in the Cox regression model, this may represent an underpowered analysis. Further studies with a larger number of rectal cancer mortality cases should be performed to evaluate the prognostic value of these methods.

Aside from being relatively simple, further investigations should consider the trichotomous TGP classification as an important prognostic predictor for colon cancer in routine pathology. Deeper molecular characterisation associated with this infiltrative pattern will undoubtedly provide novel insight into the biology that drives these morphological growth patterns.

In summary, we present the TGP scoring method, a simple and inexpensive assessment underlining the practicality in routine reporting, as an independent prognostic factor specifically for colon cancer, but not for rectal cancer or all CRC. Additionally, this method can be applied easily in routine pathological reporting without the requirement for additional processing or technologies.

## Author contributions statement

WW, CSDR, JE and NBJ contributed to the study conception and manuscript writing. WW, AM, CB, PH, KP, PA, HvW, AR, JH, NM and JP were involved in the histopathological data analysis. WW performed statistical analysis and interpretation. CSDR, JE and NBJ provided supervision throughout the study. All authors reviewed and approved the final manuscript and agreed to be accountable for the content of the work.

## Data Availability

The data for this study can be requested through an application to the Greater Glasgow and Clyde Safe Haven (GSH21ON009).
